# Stroke volume variation to guide fluid therapy: is it suitable for high-risk surgical patients? A terminated randomized controlled trial

**DOI:** 10.1186/s13741-015-0016-x

**Published:** 2015-07-22

**Authors:** Ib Jammer, Mari Tuovila, Atle Ulvik

**Affiliations:** Department of Clinical Medicine, University of Bergen, 5020 Bergen, Norway; Department of Anaesthesia and Intensive Care, Haukeland University Hospital, 5021 Bergen, Norway; Department of Anesthesiology and Intensive Care, Oulu University Hospital, PL 21, 90029 Oulu, Finland

## Abstract

**Background:**

Perioperative goal-directed fluid therapy (GDFT) may improve outcome after high-risk surgery. Minimal invasive measurement of stroke volume variation (SVV) has been recommended to guide fluid therapy. We intended to study how perioperative GDFT with arterial-based continuous SVV monitoring influences postoperative complications in a high-risk surgical population.

**Methods:**

From February 1st 2012, all ASA 3 and 4 patients undergoing abdominal surgery in two university hospitals were assessed for randomization into a control group or GDFT group. An arterial-line cardiac output monitor was used to measure SVV, and fluid was given after an algorithm in the intervention group. Restrictions of the method excluded patients undergoing laparoscopic surgery, patients with atrial fibrillation and patients with severe mitral/aortal stenosis. To detect a decrease in number of complication from 40 % in the control group to 20 % in the GDFT group, *n* = 164 patients were needed (power 80 %, alpha 0.05, two-sided test). To include the needed amount of patients, the study was estimated to last for 2 years.

**Results:**

After 1 year, 30 patients were included and the study was halted due to slow inclusion rate. Of 732 high-risk patients scheduled for abdominal surgery, 391 were screened for randomization. Of those, *n* = 249 (64 %) were excluded because a laparoscopic technique was preferred and *n* = 95 (24 %) due to atrial fibrillation.

**Conclusions:**

Our study was stopped due to a slow inclusion rate. Methodological restrictions of the arterial-line cardiac output monitor excluded the majority of patients. This leaves the question if this method is appropriate to guide fluid therapy in high-risk surgical patients.

**Trial registration:**

ClinicalTrials.gov: NCT01473446.

**Electronic supplementary material:**

The online version of this article (doi:10.1186/s13741-015-0016-x) contains supplementary material, which is available to authorized users.

## Background

Maintaining adequate oxygen supply to body organs is one of the main goals during anaesthesia, and giving intravenous fluid is one way to achieve this goal. A Cochrane systematic review found that complication rate and length of hospital stay, but not mortality, were reduced when global blood flow is optimized perioperatively by means of fluid and or drugs [1].

Recent studies show the development and use of several minimal invasive methods to estimate cardiac output and guide fluid therapy [2]. Despite the unclear evidence in the literature and contradictory findings in clinical trials, the pressure on clinicians to use a goal-directed fluid therapy approach is high. In the UK, there is even a governmental financial incentive for hospitals to use Oesophageal Doppler for its patients [3] because the goal-directed approach may be cost effective [4].

High-risk patient may have the greatest benefit of a goal-directed fluid approach [5, 6]. Less capability to compensate hypo- and hypervolemia may increase the rate of complications in a poorly optimized fluid balance [7, 8]. Benes et al. evaluate the effect of minimal invasive cardiac output-monitored fluid therapy exclusively in a high-risk abdominal surgery population [9]. Pearse describes the use of an arterial-line cardiac output monitoring in a high-risk surgery population [10]. Both studies where done on high-risk patients, using a strict definition of “high-risk”. However, we could not find a consensus in the literature about the definition of “high-risk surgery”. To simplify our approach to high-risk surgery, we defined therefore ASA 3 and 4 patients as high-risk patients. Then we intended to conduct a multicentre international prospective clinical trial to study what impact goal-directed fluid therapy based on continuous SVV (stroke volume variation) monitoring has on postoperative complications in this patient group.

## Methods

### Trial design

We planned a two-centre, assessor concealed, prospective randomized clinical trial conducted in Norway and Finland. The trial was approved by the institutional board in Norway (2011/947/REK Vest) and Finland (EETTMK:10/2012).

### Participants

From 1 February 2012 to 31 January 2013, all high-risk patients defined by ASA score 3 and 4, older than 18 years scheduled for major abdominal surgery in two university hospitals were assessed for eligibility. Patients who were able to give consent when an investigator was available were screened for eligibility. Patient undergoing liver or oesophageal surgery where not screened because they follow a more restrictive fluid regimen. Exclusion criteria after screening were the following: atrial fibrillation, severe aortic or mitral stenosis, and laparoscopic surgery or declined participation. Patients were included consecutively. Informed consent was obtained from each randomized patient.

### Interventions

Patients were randomized into two groups: a control group receiving traditional fluid therapy and a group with a goal-directed fluid therapy (GDFT) regimen guided by an arterial pressure-based cardiac output device (LiDCOrapid, LiDCO Ltd, London, UK) to measure SVV [11]. For more details of the study protocol, see Additional file 1.

### Sample size calculation

The complication rate for lower gastrointestinal surgery in elective patients in one of the study hospitals was 40 % in a previous study [12]. In the present study, a higher complication rate was expected due to inclusion of a population with a higher morbidity. To detect a decrease in number of complication from 40 % in the control group to 20 % in the GDFT group, *n* = 164 patients were needed (power 80 %, alpha 0.05, two-sided test). It was estimated that with an approximate inclusion rate of 80 patients/year in each study centre, the study could be conducted within 2 years.

### Interim analysis

Due to an unexpectedly low inclusion rate, an analysis of the exclusion factors was performed after 1 year. This resulted in termination of the study. A retrospective analysis of all patients undergoing abdominal surgery within the last year and their comorbidities and surgical techniques was undertaken after approval of the institutional board.

### Outcomes

The primary endpoint was the proportion of patients suffering of one or more complication within 5 days postoperatively.

After termination of the study, we decided to reject the primary outcome due to heavy underpowered sample size. To analyse reasons for exclusion, we determined the amount of excluded patients. No statistical analysis was performed of the numbers collected.

## Results

### Participant flow

During 1 year, *n* = 732 high-risk patients were scheduled for abdominal surgery.

Of these, *n* = 341 were not screened for inclusion. Reasons were either no investigator present or equipment missing (*n* = 224), scheduled liver or oesophagus surgery (*n* = 64) or the patient were unable to give informed consent (*n* = 53).

**Fig. 1:**
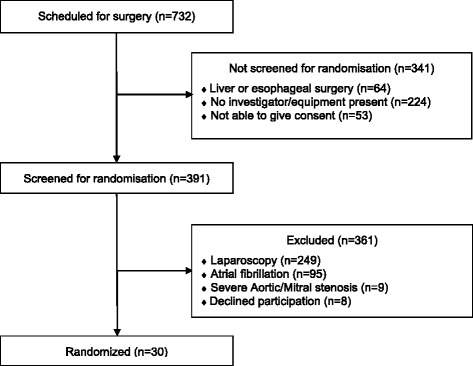
Flow diagram for patients’ progression through the trial

Of all scheduled patients, *n* = 391 patients were screened for randomisation. Of these, 64 % (*n* = 249) were excluded because a laparoscopic technique was preferred, and 24 % (*n* = 95) were excluded due to atrial fibrillation. The patient flow through the study can be seen in Fig. 1. Of all screened patients, only 7.7 % (*n* = 30) could be included in the study. The outcome data is presented by study group allocation in Table 1. A de-identified database containing all collected data of included patients is available online as an additional file (see Additional file 2).

**Table 1:** Complications (definition) within 5 days after surgery

	Intervention group	Control group
	*n* = 14	*n* = 16
Pulmonary		
Pneumonia (x-ray + antibiotics)	4	0
Pleural fluid (supplemental oxygen + x-ray)	0	2
Atelectasis (supplemental oxygen + x-ray)	3	1
Pneumothorax	0	0
Respiratory failure (intensive care treatment)	2	1
Pulmonary emboli (computed tomography + treatment)	0	0
Cardial		
Arrytmia (electrocardiogram + treatment or cardiologist consultation)	2	1
Coronary ischemia (electrocardiogram + troponin)	0	0
Pulmonary stasis/oedema (x-ray or treatment)	1	1
Neurological		
Postoperative delirium (treatment)	1	2
Focal neurological deficit	0	0
Infectious		
Wound infection (phlegmone + antibiotics or drainage)	0	0
Intraabdominal infection (computed tomography + antibiotics)	0	0
Central venous catheter infection	0	0
Wound rupture (operation)	0	0
Gastrointestinal (GI)		
Mechanical ileus (operation)	0	1
GI bleeding (transfusion or gastroscopy)	0	0
Paralytic ileus (unable to tolerate enteral diet > 5 days)	1	2
Others		
Renal impairment (creatinine increase > 33 %)	0	1
Impaired spontaneous voiding (catheterization > 2 times)	1	0
Venous thrombosis (treatment)	0	0
Sum of complications	15	12
Patients with at least one complication	7	7

### Reason for stopped trial

After 1 year, the number of randomized patients we could include in the study was only 18 % of the estimated number we have been expected at that time. We calculated that at this inclusion speed, the study would last more than 5 years and therefore decided to terminate the study early.

## Discussion

### Principal findings

In our study, a majority of high-risk surgical patients defined as ASA 3 and ASA 4 were not eligible for an arterial-line-based GDFT approach. The main reasons are methodological limitations of the arterial-line waveform analysis. The majority of patients had to be excluded because a laparoscopic surgical technique was preferred or due to atrial fibrillation.

This study was meant to be a prospective randomized controlled trial with a pragmatic approach to include patients. This would reflect daily routines, strengthening the study. Because the study was conducted in two tertiary hospitals, we had a high amount of high-risk surgical patients. Therefore, we expected to include enough patients in short time to run a well-powered study. Of 732 patients, 224 were not screened for randomisation due to investigator or equipment not being available. If this patient group also could have been screened, we may have had a higher number of patients randomized. However, it is to assume that the same fraction of patients would have to be excluded due to laparoscopic surgery and atrial fibrillation. Therefore, we do not believe that the total amount of patients that could be randomized would be much higher.

We terminated the study early, resulting in a heavily underpowered study. The primary outcome, postoperative morbidity, cannot be assessed since we just included 30 patients, and a statistical analysis would be meaningless. Consequently, we present just the patient flow numbers and not a complete statistical analysis of complications.

The low number of patients that could possibly benefit from GDFT is valid for our hospitals where the surgeons prefer to operate on high-risk patients with minimal invasive surgery. In hospitals that perform a higher amount of open surgery, the use of a minimal invasive GDFT approach may be more feasible.

We define the high-risk surgical patient by the ASA score to make the study pragmatic. However, other authors define “high-risk surgery” or the “high-risk patient” in different ways [13–15]. This makes comparison of trials dealing with this patient group difficult.

Maguire found in a retrospective electronic chart study of his hospital that *n* = 12.308 patients underwent surgery in 1 year, but only *n* = 4.792 (39 %) fulfilled the criteria for an arterial-line-based cardiac output monitor, and of these, only 23.2 % had an arterial-line. There was no report on how many of the patients were ASA III/IV patients [16].

Arterial-line-based waveform analysis measures hemodynamics by calculation of stroke volume variation or pulse pressure variation. However, arterial-line-based output methods are not applicable to large patient groups due to their limitations [16]. One limitation is laparoscopic procedures [17]. The increased intraabdominal pressure from the pneumoperitoneum affects dynamic parameters independently in changes of volume status [17–19]. Consequently would SVV during pneumoperitoneum increase while the blood volume do not decrease, it would lead to false positive readings [20]. It is therefore not well validated in humans [21, 22]. Other limitations of waveform analysis measurements are cardiac arrhythmias and patients with severe cardiac valvulopatias [23].

Despite criticism about the evidence of the effect of goal-directed therapy, one single method of minimal invasive cardiac output monitoring (Oesophagus Doppler) has even been officially recommended in the National Institute for Health and Clinical Excellence guidelines of the UK (http://www.nice.org.uk/guidance/MTG3). This decision has been criticized due to the lack of proof [24, 25], and the method may not be superior to a strategy of a neutral balance [26]. Other studies have not found benefices in a goal-directed fluid approach [10, 12, 26–30], have not found benefit using a restrictive fluid approach [31] or even have worse outcome in physically fit patients [5].

It is biologically plausible that the right amount of fluid given at the right time increase oxygen delivery to the organs and thereby benefit patient outcome. There has been a meta-analysis confirming that a GDFT approach may decrease postoperative complications. However, many included studies are small single centre studies with a high risk of bias or methodological limitations [1, 32, 6]. The effect on outcome in these studies is mostly small. An even statistical distribution of different studies with a small effect size would consequently result in a number of studies that would show no effect or even harm. The marked overweight of studies with a small positive effect on outcome may indicate a publication bias favouring trials with positive results. This may mask limitations of the arterial-line-based GDFT method that we report. Other studies investigating high-risk surgical patients do not report the exclusion rate due to atrial fibrillation when this condition restricted the GDFT method used [5, 9].

The OPTIMIZE trial with a study population of 734 patients is the largest trial on GDFT to date. It could not show a reduction of complications after perioperative arterial-line-based GDFT. However, when including the OPTIMIZE trial in an updated Cochrane meta-analysis, it indicates a reduced complication rate [10].

Goal-directed fluid therapy may be more important in a high-risk surgery population than in a relatively healthy population. Limitations of the method with an arterial-line-based monitor may cause exclusion of a patient group who may benefit most of the treatment. In the UK, it is recommended to use an Oesophagus Doppler to guide fluid therapy preoperatively. The same limitations that apply to the arterial-line-based method (exclusion of patient with atrial fibrillation and laparoscopic procedures) would apply to this method too.

## Conclusions

Our primary goal was to investigate if high-risk surgical patients benefit from SVV-guided fluid therapy. This question still remains open. A majority of our patients had to be excluded from the trial due to methodological limitations. This leaves the question whether or not an arterial-line-based cardiac output monitor is the best method to guide fluid therapy in high-risk surgical patients.

## Additional files

Additional file 1:
**Study protocol.** Study protocol, Word 2010 document. Additional file 2:
**De-identified SPSS database containing collected data of all randomized patients.** SPSS Statistics Data Document.

## Abbreviations

ASA: American society of anesthesia score
GDFT: Goal-directed fluid therapy
SVV: Stroke volume variation
