# Supplementary material for: Stroke volume variation to guide fluid therapy: is it suitable for high-risk surgical patients? A terminated randomized controlled trial
Source: Perioper Med (Lond). 2015 Jul 22;4:6. doi: 10.1186/s13741-015-0016-x (PMC4511544; doi:10.1186/s13741-015-0016-x)
Supplement: Additional file 1: — Study protocol. Study protocol, Word 2010 document. [file 13741_2015_16_MOESM1_ESM.doc]

Protocol

Title: Traditional versus goal directed perioperative fluid therapy in high risk patients. A randomized, assessor-blinded study.

Background

One of the goals during anaesthesia is to maintain adequate oxygen supply to body organs. Anaesthetic agents provoke vasodilatation and a varying degree of myocardial depression. The blood flow for each organ is not known clinically and the measurement of cardiac volume requires invasive methods or expensive equipment and an experienced investigator. It is therefore common to use "surrogate parameters". One of these is hourly urine output which is recommended to be kept above 0.5-1ml / kg / h. However, there are no studies showing that low peroperativ urine output predict postoperative kidneyfailure. A Cochrane review from 2008 found no evidence for the efficacy of perioperative renalprotective measures (Zacharias, Conlon et al. 2008), whether as drugs, nor as intravenous fluids.

Since several physiological mechanisms during anaesthesia and surgery prevents the production of urine (aldosterone, glucocorticoids, ADH), there is often provided lots of fluid to maintain a urine flow of 0.5 ml / kg / h. Electrolyte-containing fluids are distributed throughout the whole extracellular matrix. Attempts in filling the circulation system with a liquid where only 1/5^th^ - 1/4^th^ remains, must necessarily lead to a salt and fluid overload. Several litre of excess fluid causes oedema.

In recent years, this regime has been criticized. Fluid restriction was part of the protocol for "fast-trach" gastrointestinal surgery (Basse, Hjort Jakobsen et al. 2000) with the reason that ample hydration is unfavourable and increases the complication rate after surgery (Holte, Sharrock et al. 2002). This view has been supported by two randomized, controlled trials on a gastro surgical patient group (Brandstrup, Tonnesen et al. 2003; Nisanevich, Felsenstein et al. 2005).

Simultaneously with these studies indicating the benefits of limiting perioperative fluids, there have been published studies that suggest that individualized and goal directed fluidsupply can reduce the complication rate and length of hospital stay after surgery. Shoemaker showed that supra-normal oxygen transport was associated with improved survival in high risk surgical patients (Shoemaker, Appel et al. 1988). A large meta-analysis from 2005 (Poeze, Greve et al. 2005) concluded that hemodynamic optimization perioperatively is leading to lower mortality.
They are also reports of beneficial effects of goal directed fluid therapy in elective gastro surgical patients (Wake Ling, McFall et al. 2005; Noblett, Snowden et al. 2006). Common to the intervention group in these studies is that the fluid provided optimize the cardiac volume, as estimated on the basis of the measured Doppler flow in the aorta descends. The intervention group received a mean of same or greater fluid amount than the control group, but there was a large individual spread. Measuring cardiac output perioperatively is complicated. Oesophagus Doppler is little invasive, but requires training and costs a lot.

Optimizing fluid therapy guided by central venous oxygen-saturation showed no difference in postoperative morbidity compared with traditional fluid therapy (Jammer, Ulvik et al. 2010). The reason for this may be a relatively healthy group of patients who tolerate excess fluid. Fluid optimization to prevent postoperative complications is still considered to be important for very ill and old patients. These have less compensation opportunities when hypo-and hypervolemic and are therefore more susceptible to complications in a poorly optimized fluid balance.

An alternative method for guiding optimization of fluid therapy is the measurement of stroke volume variation (SVV): Under anaesthesia, the patient is ventilated with positive pressure. This changes the intra-thoracic pressure in line with the respiratory rate. The pressure in the right atrium increases during inspiration and thus decreases venous return flow, which in turn leads to lower stroke volume. During expiration the pressure and stroke volume increases. On the surveillance monitor, the difference in stroke volume between inspiration and expiration is recorded as an oscillation in the arterial pressure curve. This oscillation is called Stroke Volume Variation (SVV). It is well known for over 20 years that SVV can be used to assess volume status (Cannesson, Aboy et al. 2010). When the intravascular blood volume is low, the oscillation is greater (SVV> 10%). When giving a fluid bolus, stroke volume increases and thus reduces the oscillation in the blood pressure curve and SVV (Mayer and Suttner 2009; Cannesson, Aboy et al. 2010). This principle for fluid optimization has been used in several studies (Benes, Chytra et al. 2010; Mayer, Boldt et al. 2010; Davies, Yates et al. 2011).

To calculate SVV the stroke volume (SV) is measured through an arterial line. Arterial access is standard procedure in ASA 3-4 patients. Fluid optimization is therefore possible without adding additional invasive procedure for the patient.

Two types of monitors available to measure SV through an arterial line: FloTrac/Vigelio (Edwards Scientific, Irvine, USA) and LiDCO monitor (LiDCO Group, London, United Kingdom) (Mayer and Suttner 2009). FloTrac/Vigelio analyze the morphology of the pulse wave curve and estimate SV from a normogram and then calculate SVV. The formula for the calculation of SV was updated several times in recent years due to problems with accuracy. In addition, the method is prone to measurement errors in arrhythmia, poor pulse curve or vasopressor use (Eleftheriadis, Galatoudis et al. 2009; Slagt, Beute et al. 2010; Metzelder, Coburn et al. 2011). LiDCOrapid from LiDCO, London, United Kingdom calculates the area under the pulse curve (Area Under the Curve, AUC) for the estimation of SV using a normogram. The calculation algorithm is unchanged since the invention. It has been tested repeatedly and found to be equivalent to an established method for the measurement of SV (de Wilde, Schreuder et al. 2007). In our study, we measure SV and SVV using the LiDCOrapid monitor.

Through the arterial line, blood samples are taken and base excess as well as lactate will be measured pre-and postoperatively. Lactate is used as a marker of poor tissue perfusion (More Galli, Oliveira et al. 2004), while base excess is a good marker of survival in trauma patients (Kroezen, Bijlsma et al. 2007).

Purpose / hypothesis
**Is goal directed fluid therapy reducing postoperative complications in comparison to traditional fluid therapy for gastro surgical ASA III / IV patients?**

We compare two groups of patients: one group receives goal directed fluid therapy guided by LiDCOrapid stroke volume variation (SVV), the other gets the "traditional" fluids, ie the current regime.

Design
Prospective, multicenter, partially blinded, randomized study.

Selection
Inclusion criteria:
Adult ASA class III & IV (high risk) patientsm, >18 years, scheduled for gastrointestinal surgery involving laparotomy. Both elective and emergency cases are included.

Exclusion Criteria:
Atrial fibrillation
Mental impairment, unable to give informed consent
Severe aortic or mitral stenosis
Type of surgery: the liver, transthoracic oesophagectomy

Intervention:

Patients are randomized into a control group and an intervention group. The control group receives fluid therapy by today's standards, the intervention group receives goal directed fluid therapy guided by stroke volume variation (SVV).

Primary outcome:
Postoperative complications within 5 days, see table.

Secondary outcome

- Renal function: defined by RIFLE criteria. (Cruz, Bagshaw et al. 2010)
- Lactate, base excess and PaO2: difference between preoperative and postoperative values ​​in the two study groups.
- Urine Production: Amount of urine per-and postoperatively until the patient is sent to ward..
- Length of hospital stay
- Complications until discharge and readmission within 30 days.
- Vasopressor needs: Differences in the number of patients who need vasopressor (noradrenalin) during and after surgery.
- Mortality: within 30 days and 3-month after surgery in both groups.

Power Calculation:
The complication rate for lower gastrointestinal surgery in elective patients on Haukeland was 40% in a previous study (Jammer, Ulvik et al. 2010). It is expected to reach a higher complication rate because patients in this study are sicker. We want to detect clinically relevant complications. Complications of diagnostic criteria can be found in the attached table.
Based on the primary outcome (number of patients with complications), 82 patients need to be included in each group. This in order to detect a decrease in the number of patients with complications from 40% in the control group to 20% in the intervention group.

Power 80%, alpha = 0.05, two-sided test
**Total should 164 patients being included.**

Randomization
Randomisation takes place in the operating room after informed consent was obtained. Randomization is performed when it is certain that the patient truly is going to be operated. Stratification is done for degree of surgery (emergency, elective) and the hospital. Registration is done by use of an Internet-based solution developed by Unit for Applied Clinical Research (AKF) and IT department at NTNU/Trondheim. Those who provide the data are getting a login with username and password. The password is stored in encrypted form on the server. Patient data is stored as initials and age or year of birth, which also is encrypted. Direct person-identifiable data is replaced with a reference number that refers to a list of names. This list is kept at the center that includes the person in the study, separate from the rest of the data. AKF has no access to the list of personally identifiable information. The server with the database is continuously monitored and upgraded on an ongoing basis with the latest safetyupdates. All data is backed up once per day.

Blinding:
Morbidity data is collected by a study physician who does not know the randomization. Patients are being visited 5 days after surgery or earlier when patient is being discharged before that. Morbidity is being recorded then.

Mortality data, length of hospital stay and morbidity data until discharge is obtained from the patient record 3 months after surgery.

Anesthesia Method
Bowel Emptying on elective patients the day before surgery is determined by the surgeon. Patients can drink clear liquids until 2 hours before surgery. This does not apply to emergency patients with ileus. Before arrival in the operating room the bladder is being emptied. Insertion of epidural catheter in the lower thoracic area. The catheter is being tested and the patient receives continuous epidural infusion of bupivacaine 1 mg/ml, fentanyl 0.002mg/ml and adrenaline 0,002 mg/ml, usually 8 ml/h which can be adjusted per and postoperatively if necessary. Standard monitoring, including continuous ECG, invasive blood pressure, capnography and pulsoxymetry. Blood gas is taken and recorded. Induction of anaesthesia with thiopental, propofol or etomidate; fentanyl and vecuronium. Maintenance of anesthesia with isoflurane, desflouran or sevoflouran at MAC 0,7-1,3 and fentanyl. Nitrous oxide is not used.
Inspiratory oxygen (FiO2) is increased above 35% if necessary to have SpO2 ≥ 95%. To get the correct measurement of the LiDCO monitor, tidalvolum is kept above 8 ml / kg and respiratory rate adjusted to pCO2 30-37mmHg with PEEP between 0 and 5cmH2O (Cannesson 2009). Patients are being warmed active with warm intravenous fluids and warm touch. Body temperature is kept above 35.5 ° C. Low molecular weight heparin given as thromboprophylaxis. Blood is usually given if blood loss >1000ml, look otherwise at table for fluid therapy (p. 9). Central venous catheters placed as required by the anesthetist's assessment. Postoperative fluid balance is recorded up to 24 hours after surgery start.

Fluid therapy:
For fluid treatment, adjusted body weight (lean body weight + 1/3 of overweight) is used.

Intervention group: Standard monitoring. The patient is connected to the LiDCOrapid monitor via an arterial line placed in a.radialis. A fluidbolus (medical fluid after surgical prescription (antibiotics: metronidazole 300ml, doxycykline 500ml), or Ringer acetate until the patient has received 800ml liquid) is given before anesthesia. If the stroke volume (SV) increases more than 10%, repeat the procedure with 6ml/kg Ringer acetate until the SV is not increasing. After that, induction of anesthesia.
Maintenance fluid is given as Ringer acetate 2ml/kg/t. Continuous monitoring of stroke volume variation (SVV). If SVV> 10%, give a fluid bolus 6ml/kg Ringer acetate. Repeat until SVV <10%. Bleeding is being replaced 1:1 with hydroxyethyl starch. SAG by bleeding >1000ml. By fall in blood pressure and SVV <10%, start vasoactive treatment with noradrenalin (Hiltebrand, Koepfli et al. 2011). Postoperative is given Glucose 5% 80ml/h. HES or Ringer when blood pressure low, eventually noradrenaline as vasoactive agent.

Control group:
Patients receive preoperative medical fluid after surgical prescription (antibiotics: metronidazole 300ml, doxycykline 500ml), or Ringer acetate until the patient has received 800ml liquid. Standard monitoring. Initial optimization of fluid status is performed by pulse, BP and anaesthesiologist assessment with Ringer acetate. Followed by an infusion of 8-10ml/kg/t Ringer acetate the first hour and 6-8ml/kg/t the following hours. Urinary output and blood pressure is used as a surrogate parameter: the infusion rate is increased by a fall in blood pressure or urine output <0.5ml/kg/t. Bleeding replaced with HES 1:1, otherwise see table for fluid therapy page 9. Vasoactive agents (noradrenaline / phenylephrine) is given if the anesthesiologist considers this necessary. Postoperative give 1000ml Glucose 5%. HES or Ringer when low blood pressure, eventually noradrenaline as vasoactive agent.

Data Acquisition
Circulation Data and data on patients' health status and functional level is recorded on a paperbased case record form (CRF). It is then transferred to an secure internet based webside maintenanced by the Unit for Applied Clinical Research, NTNU/Trondheim.

The data set collection includes:
Randomisation data
• Name
• Birthdate
• Gender
• Patient Number
• ASA grades
• P-Possum scores
• Urgent rate (elective / emergency)
• Type of surgery
Baseline data
• Diagnosis
• Type of anesthesia
• Weight / height

• Adjusted body weight (lean body weight + 1/3 of overweight)

• Preoperative functional status (Karnofsky index)
• Comorbidity

o Coronary Disease

o Heart Failure

o COPD / Asthma

o Diabetes mellitus with insulin therapy

o Diabetes mellitus without insulin therapy

o Cancer

o Stroke or TIA (transient ischemic attack)

o use of anticoagulantia

• duration of surgery
• Creatinine and GFR before surgery and 3 days after.
• Lactate, BE, Na, K and PaO2 prior to surgery, after completion of surgery, and 12-18 hours after surgery.
• The need for postoperative intensive care

Fluid
• Amount of fluid, given perioperatively and postoperatively (colloid/crystalloids/ glucose)
• Other fluids (SAG, medical fluid)
• Amount of bleeding
• Urine production
• fluidloss via stoma
Medications
• Use of vasoactive agents peroperativt
• Use of vasoactive agents postoperative
• Post-operative epidural analgesia
Clinical outcome
• 5-day postoperative morbidity
• 30-day postoperative mortality
• 30-day postoperative morbidity and readmission
• 90-day postoperative mortality
• Length of stay (LOS, Length of hospital stay)

The patient's anesthesia record shall not include randomization or data that may indentify randomisation. Total fluid administered is recorded in the anaesthesia record as usual. Morbidity registration is done on day 5 after surgery by visiting the ward and after 3 months of data from the patients chart. Data is recorded on the case record form and then transferred to the database.

Resources It sought funding from various grants and internal assets.

Schedule
Time for realisation of the study will depend on whether other hospitals participate. It is realistic to include about 70 patients per year at Haukeland.

Publication
The aim is publishing in an English-language journal.
Responsible for the study is Ib Jammer, physician, Atle Ulvik, PhD, attending and Gro Østgaard, PhD, chief attending anesthesia, KSK, Haukeland University Hospital.

| In the operation theatre | Traditional (dept. routine) | Intervention group |
| --- | --- | --- |
| Antibiotics (Metronidazol 300ml, Doxycycline 500ml)  Induction  Maintenance  SVV  Bt mean <60mmHg >10 min without response to adjustment of anesthetic depth or other measures  Replacement of blood loss  Acute major bleeding  Hb < 8 ongoing bleeding  Hb < 7 (or 9, coronary/age)  Urinary output <0.5ml/kg/h >3t | either (500 + 300 antibiotics), or 800ml Ringer  Ringer 1000ml or after anaesthesiologists assessment  Ringer 10ml/kg/t via  infusion pump    Is not measured  Fluid bolus with Ringer by anaesthesiologists’ assessment. Eventually metaoxedrin or noradrenalin infusion  Bleeding until 1000ml =>1:1 HES, thereafter SAG  >1000ml = SAG  SAG  SAG  Bolus Ringer | either (500 + 300 antibiotics), or 800ml Ringer  Connection of LiDCOrapid via Aterial line. Measuring of SV. 500ml Ringer before induction. If SV- increase >10% repeat bolus until SV- increase <10%  Ringer 2ml/kg/t via infusion pump  Is measured with LiDCO during operation.  (follow algorithm, bolus with 6ml/kg Ringer if SVV>10%)  If SVV<10% noradrenalin infusion  Bleeding until 1000ml =>1:1 HES, thereafter SAG  >1000ml = SAG  SAG  SAG  No action |

| Postoperative (until 24h after surgery) | Traditional (dept. routine) | Intervention group |
| --- | --- | --- |
| Maintenance  Bt mean < 60mmHg > 15 min  Urinary output < 0,5ml/kg/t > 2t | Eventually rest Ringer,  1000ml Glucose 5%, evt. + KCl  Ringer, evt HES  Evt vasoactive agents (efedrin sc or noradrenalin infusion)  Ringer | Fluid p.o.  Glucose 5%, evt + KCl ca 80ml/t  Ringer for compensation of loss through stoma    Ringer, evt HES  Evt vasoactive agents (efedrin sc or noradrenalin infusion)  No action |
| Fluidtreatment when discharge to ward on day 1 | Fluid p.o., evt glucose 5% 1000ml, evt Ringer for compensation of loss through stoma | Fluid p.o., evt glucose 5% 80ml/t evt Ringer for compensation of loss through stoma |

|  | Definition of complications |
| --- | --- |
| Respiration |  |
| Pneumonia | X-ray + antibiotics (AB) |
| Pleuraeffusion | Dyspnea in need of extra O2 + X-ray |
| Atelectasis | Dyspnea in need of extra O2 + X-ray |
| Pneumothorax | X-ray |
| Respiratory Failure | Treatment at intensive care or lung ward |
| Pulmonary embolism | CT, anticoagulation |
| Cardiovaskular |  |
| Arrhythmias | ECG + medical supervision and / or treatment |
| Coronarischemia/ infarct | ECG changes and / or > troponin |
| Pulmonary congestion / edema | X-ray and / or treatment |
| CNS |  |
| Postoperative confusion | Treatment with psychotropic drugs |
| Focal neurological symptoms | clinics and CT |
| Infection |  |
| Wound infection | antibiotics + phlegmone and / or drainage |
| Intra-abdominal infection / Anastomosislekage | antibiotics + CT, ultrasound or surgery |
| Central Venous Catheter infection | antibiotics |
| Wound rupture | Operation |
| Gastrointestinale |  |
| Mechanical ileus | Operation |
| GI bleeding | Transfusion, evt skopy |
| Paralytic ileus | > 5dgr without food tolerance |
| Kidney / urinary tract |  |
| Postoperative renal failure | Creatinine> 50% increase from preoperatively or <0.5 ml / kg / h of urine over 6 hours |
| Lack of spontaneous urination | Catheterization >2 times |
| Venous thrombosis | drug treatment |

Algorithm fluids, the intervention group

If bloodpressure fall, evt vasoactive agents

6ml/kg Ringer in

10min

wait 5 min

SVV fall?

SVV >10%

yes

no

no

 

yes

Basse, L., D. Hjort Jakobsen, et al. (2000). "A clinical pathway two Accelerate recovery after colonic resection." Ann Surg 232 (1): 51-57.

Benes, J., I. Chytra, et al. (2010). "Intraoperative fluid optimization overusing stroke volume variation in high risk surgical patient: results of prospective randomized study." Critical Care 14 (3): R118.

Brandstrup, B., H. Tonnesen, et al. (2003). "Effects of intravenous fluid restriction Wed postoperative complication: comparison of two perioperative fluid regimens: a randomized assessor-blinded multicenter trial." Ann Surg 238 (5): 641-648.

Cannesson, M. (2009). "Arterial Pressure Variation and Goal-Directed Fluid Therapy." J Cardiothorac Anesth Vasca.

Cannesson, M., M. Aboy, et al. (2010). "Pulse pressure variation: where are we today?" Journal of clinical monitoring and computing.

Cruz, D. N., S. M. Bagshaw, et al. (2010). "Acute kidney injury: classification and staging." Contributions two Nephrology 164: 24-32.

Davies, S. J., D. Yates, et al. (2011). "Dopexamine ha no additional benefit in high-risk Patient Receiving goal-directed fluid therapy under going major abdominal surgery." Anesth Analg 112 (1): 130-138.

de Wilde, R. B., J. J. Schreuder, et al. (2007). "An evaluation of cardiac output by five arterial pulse contour techniques hum cardiac surgery." Anaesthesia 62 (8): 760-768.

Eleftheriadis, S., Z. Galatoudis, et al. (2009). "Variations in arterial blood pressure are associate with parallel changes in FlowTrac / Vigileo-derived cardiac output measurements: a prospective comparison study." Critical Care 13 (6): R179.

Hiltebrand, L. B., E. Koepfli, et al. (2011). "Hypotension hum Fluid-restricted Abdominal Surgery: Effects of norepinephrine Treatment Wed Regional and Microcirculatory Blood Flow in the Intestinal Tract." Anesthesiology 114 (3): 557-564.

Holte, K., N. E. Sharrock, et al. (2002). "Pathophysiology and Clinical Implications of perioperative fluid excess." Br J Anaesth 89 (4): 622-632.

Jammer, I., A. Ulvik, et al. (2010). "Does central venous oxygen saturation-directed fluid therapy Affect postoperative morbidity after colorectal surgery? A randomized assessor-blinded controlled trial." Anesthesiology 113 (5): 1072-1080.

Kroezen, F., T. S. Bijlsma et al. (2007). "Base deficit-based predictive modeling of outcome in trauma patient admitted two intensive care units in Dutch trauma centers." The Journal of Trauma 63 (4): 908-913.

Mayer, J., J. Boldt, et al. (2010). "Goal-directed intraoperative therapy based on autocalibrated arterial pressure waveform analysis reducers hospital stay in high-risk surgical patient: a randomized, controlled trial." Crit Care 14 (1): R18.

Mayer, J. and S. Suttner (2009). "Cardiac output derived from arterial pressure waveform." Curr opine Anaesthesiol 22 (6): 804-808.

More Galli, A., R. P. Oliveira, et al. (2004). "Occult hypoperfusion is Associated with Increased Mortality in hemodynamically stable, high-risk, surgical patients." Critical Care 8 (2): R60-65.

Metzelder, P., M. Coburn, et al. (2011). "Performance of cardiac output measurement derived from arterial pressure waveform analysis in patient requiring high-dose vasopressor therapy." British Journal of Anaesthesia.

Nisanevich, V., I. Felsenstein, et al. (2005). "Effect of intraoperative fluid management Wed outcome after intra-abdominal surgery." Anesthesiology 103 (1): 25-32.

Noblett, S. E., C. P. Snowden, et al. (2006). "Randomized clinical trial Assessing the effect of Doppler-optimized fluid management Wed outcome after elective colorectal resection." Br J Surg 93 (9): 1069-1076.

Poeze, M., J. W. Greve, et al. (2005). "Meta-analysis of hemodynamic optimization: relationship two methodological quality." Crit Care 9 (6): R771-779.

Shoemaker, W. C, P. L. Appel, et al. (1988). "Prospective trial of supra-normal values ​​of survivors as Therapeutic goals in high-risk surgical patients." Chest 94 (6): 1176-1186.

Slagt, C., J. Beute, et al. (2010). "Cardiac output derived from arterial pressure waveform analysis without calibration Vs. Thermodilution in septic shock: evolving Accuracy of software versions." European journal of anaesthesiology 27 (6): 550-554.

Tote, S. P. and R. M. Grounds (2006). "Performing perioperative optimization of the high-risk surgical patient." Br J Anaesth 97 (1): 4-11.

Wake Ling, H. G., M. R. McFall, et al. (2005). "Intraoperative oesophageal Doppler guided fluid management shortens postoperative hospital stay after major bowel surgery." Br J Anaesth 95 (5): 634-642.

Zacharias, M., N. P. Conlon, et al. (2008). "Interventions for Protecting renal function in the perioperative period." Cochrane Database of Systematic Reviews (4): CD003590.
